# Ergebnisse zur Reduzierung von subkutanem Fettgewebe mithilfe eines neuartigen, auf Mikrowellentechnologie basierenden Body-Contouring-Systems

**DOI:** 10.1007/s00105-023-05200-y

**Published:** 2023-08-28

**Authors:** Paolo Bonan, Federica Coli

**Affiliations:** 1 Abteilung Laser/Plastic Surgery, Universität Siena, Siena, Italien; 2 Villa Donatello Clinic, Viale G. Matteotti, 4, 50132 Florenz, Italien

**Keywords:** Lokale Fettansammlung, Cellulite, Bauchfalte, Bauchumfang, Hautstraffung, Localized fat, Cellulite, Adominal fold, Abdominal circumference, Skin tighthening

## Abstract

**Hintergrund:**

Im vergangenen Jahrzehnt hat der Body-Shaping-Markt kontinuierliche Zuwächse verzeichnet. Um die Verfahren zur Reduzierung von lokalen Fettansammlungen zu verbessern, wurde eine neuartige Methode entwickelt, die sich speziell auf subkutanes Körperfett konzentriert.

**Studienziel:**

Ziel dieser Studie ist es nachzuweisen, dass ein neuartiges Mikrowellengerät mit einer Frequenz von 2,45 GHz bei der Reduzierung von Fettzellen sichtbare Ergebnisse mit größerer Sicherheit und Beständigkeit erzielt.

**Materialien und Methoden:**

Neunzehn gesunde Patienten – 10 Frauen und 9 Männer – (im Alter von 24 bis 55 Jahren, was einem Altersdurchschnitt von 39 Jahren entspricht) und sichtbaren Fettansammlungen im Bauchbereich erhielten 3 Mikrowellenbehandlungssitzungen (im Abstand von jeweils 4 Wochen) unter Verwendung des neuen Onda Plus Body Shaping-Systems (von DEKA, Italien). Dieses Gerät verwendet einen Mikrowellenapplikator mit einer Frequenz von 2,45 GHz und einer integrierten Kühlung für einen optimalen Behandlungskomfort des Patienten während der Anwendung. Es wurde ein geeignetes Behandlungsprotokoll festgelegt, das ca. 10 min lang in jedem Behandlungsbereich angewandt wurde. Vor Beginn der Behandlung haben alle Patienten eine Einwilligungserklärung sowie ein Fotofreigabeformular unterzeichnet. Vor jeder Behandlungssitzung wurden folgende Daten erfasst: Körpergewicht, Größe, Taillenumfang sowie Fotos. Alle zu behandelnden Bereiche wurden im Stehen mit einem hautverträglichen weißen Stift vorgezeichnet. Ausgeschlossen wurden adipöse Patienten und solche, bei denen die Fettablagerungen über den gesamten Körper verteilt waren oder deren Haut sich in einem Zustand irreversibler Schlaffheit befand. Bei jedem Patienten wurde eine Blutuntersuchung durchgeführt – sowohl vor Beginn der Behandlung (T0) als auch am Ende des gesamten Behandlungsprotokolls (T3).

**Ergebnisse:**

Sämtliche Patienten erfüllten die Einschluss‑/Ausschlusskriterien der Studie und unterzeichneten eine Einwilligungserklärung. Die 19 gesunden Erwachsenen wurden in 3 Gruppen aufgeteilt je nach Größe der Bauchfalte (Pinch). Gruppe 1 bestand aus 4 Patienten mit einem Pinch von mehr als 4,5 cm, Gruppe 2 bestand aus 10 Patienten mit einem Pinch von 2,5–4,5 cm, und Gruppe 3 bestand aus 5 Patienten mit einem Pinch von weniger als 2,5 cm. Die Ergebnisse zeigten, dass beim Follow-up nach 3 Monaten die klinisch messbare Reduzierung des Bauchumfangs bei allen Patienten 3,80 ± 1,21 cm betrug.

**Schlussfolgerung:**

Es hat sich gezeigt, dass das mikrowellenbasierte Body-Contouring-System sicher und effektiv für die Reduzierung des Bauchumfangs verwendet werden kann. Es wurden weder starke Schmerzen noch merkliches Unbehagen während einer der Behandlungssitzungen berichtet.

## Abstract (en)

**Background:**

Over the past decade, the body shaping market has seen steady growth. In order to improve procedures to reduce localized fat deposits, a novel method specifically targeting subcutaneous adipose tissue was developed.

**Objective:**

The objective of this study is to demonstrate that a novel microwave device with a frequency of 2.45 GHz achieves visible results with greater safety and consistency in reducing adipose tissue.

**Materials and methods:**

In all, 19 healthy patients (10 women and 9 men; age 24–55 years, average age 39 years) and visible abdominal fat received three microwave treatment sessions (each 4 weeks apart) using the new Onda Plus Body Shaping System (DEKA, Italy). The device uses a handpiece that generates microwaves with a frequency of 2.45 GHz and integrated cooling for optimal patient comfort during treatment. An appropriate treatment protocol was established and applied for approximately 10 min in each treatment area. Before the start of treatment, informed consent and photo release forms were signed by all patients. Before each treatment session, the following were collected: body weight, height, waist circumference, and photographs of the area to be treated. All areas to be treated were premarked with a white skin marker pen while standing. Obese patients and those in whom fat deposits were distributed over the entire body or whose skin was in a state of irreversible flaccidity were excluded. A blood test was performed for each patient, both before the start of treatment (T0) and at the end of the entire treatment protocol (T3).

**Results:**

All patients met the inclusion/exclusion criteria of the study and signed an informed consent form. The 19 healthy adults were divided into three groups according to the size of the abdominal fold (pinch): group 1 comprised 4 patients with a pinch greater than 4.5 cm, group 2 had 10 patients with a pinch of 2.5–4.5 cm, and group 3 had 5 patients with a pinch less than 2.5 cm. At the 3‑month follow-up, the clinically measurable reduction in abdominal circumference was 3.80 ± 1.21 cm in all patients.

**Conclusion:**

The microwave-based body contouring system was shown to be safe and effective to reduce abdominal circumference. No severe pain or noticeable discomfort was reported during any of the treatment sessions

Der Body-Shaping-Markt hat in den vergangenen 10 Jahren einen kontinuierlichen Zuwachs verzeichnet. Dies ist zurückzuführen auf die permanente Nachfrage nach nichtchirurgischen und nichtinvasiven Behandlungsformen bei Fettansammlungen und Cellulite sowie zur Hautstraffung [1]. Heutzutage steht eine ganze Palette unterschiedlicher Methoden zur Verfügung, um die Körperkontur zu optimieren. Auch wenn einige dieser Methoden und Geräte eine gewisse Wirksamkeit auf Fettzellen oder Cellulite haben, steigt zunehmend der Bedarf nach einem Kombinationsgerät, das nicht nur Wirksamkeit und Sicherheit gewährleistet, sondern auch eine geringe Anzahl von Sitzungen für alle Anforderungen des Body Contouring benötigt [2, 3]. Angesichts dieser Tatsache ist die moderne Technologie auf der Suche nach neuen Lösungen, um medizinischen Anwendern multiple Plattformen zu liefern, die ein möglichst hohes Level an Patientenkomfort bieten.

Die Mikrowellen(MW)-Technologie wird in der modernen Welt auf vielfältige Weise genutzt – sowohl im Alltag als auch in vielen Bereichen der Medizin bis hin zur Onkologie, Chirurgie und Dermatologie. Nach umfassenden Studien [4, 5] geht man davon aus, dass die Anwendung nichtinvasiver hochenergetischer 2,45-GHz-Mikrowellen [6] in Körperbereichen mit Fettansammlungen fokussiert auf das subkutane Fettgewebe wirkt und so die Erhitzung der Fettzellen (Adipozyten) stimuliert, ohne dass die darüber liegenden epidermalen und dermalen Schichten davon betroffen werden. Dies hat eine metabolisch vollständig kompatible Makrophagenlipolyse mit Reduzierung des subkutanen Fettgewebes zur Folge. Da Cellulite durch die Solubilisierung der Kollagensepten innerhalb der Cellulite verbessert wird, verursacht die Erhitzung des subkutanen Fettgewebes ein Zusammenziehen der dermalen Kollagenfasern und verbessert so die externe Architektur der Haut.

Der Grund für die Verwendung von Mikrowellen zur Reduzierung von subkutanem Fett liegt in deren selektiver Wirkung auf die Fettzellen bei der Anwendung auf Hautgewebe [6]. MW sind Ausdruck eines elektromagnetischen Felds, und wenn sie auf biologisches Gewebe angewendet werden, verursachen sie gewisse Oszillations- und Schwingungsphänomene in den Hautmolekülen, auf die sie angewendet werden. MW werden als sicher eingestuft, weil sie sich signifikant abmildern, bevor sie innere Organe erreichen könnten. Diese Eigenschaften machen sie geeignet für Behandlungen zur Fettreduzierung [6]. Nachweislich ist eine Frequenz von 2,45 GHz geeignet, um ohne substanziellen Verlust der Energiedichte tiefes subkutanes Gewebe zu erreichen [6]. Es hat sich gezeigt, dass bei einer solch hohen Frequenz das Hautgewebe fast „transparent“ bei der Durchleitung der Energie ist, die fast vollständig insbesondere über die subkutane Fettschicht geleitet wird. Dadurch werden die oberflächlichen Schichten der Dermis vor unerwünschter Erhitzung geschützt und Verbrennungen vermieden.

Mikrowellen gelten als sicher, weil sie sich signifikant abmildern, bevor sie innere Organe erreichen

Betrachtet man verschiedene Frequenzen (von Radiofrequenz bis hin zu Mikrowellen) hinsichtlich ihrer Wirkungsweise in der Haut, kann man feststellen, dass ein Großteil der elektromagnetischen Energie in der Epidermis und Dermis verbleibt und nicht in die subkutane Fettschicht vordringt. Diese Energien erhitzen die dermoepidermale Schicht so weit, dass das Risiko von Gewebeschäden besteht. Im Gegensatz zur Radiofrequenzenergie, die größtenteils nahe der Oberfläche verbleibt, erreichen diese MW gezielt die Hypodermis, in der sich die Fettzellen befinden, und sorgen so für eine effektive Behandlung.

Wenn sie die subkutane Schicht erreichen, fördern die 2,45-GHz-MW die Freisetzung überschüssiger, nicht veresterter freier Fettsäuren in Form kleiner Tröpfchen oder Bläschen, die sich nach und nach von der Oberfläche der Fettzelle in Richtung Interstitium ablösen. Der Transport solcher Fetttröpfchen, die morphologisch deutlich unter dem Elektronenmikroskop zu erkennen sind [7], nach außerhalb des Adipozyten wird als „Blebbing“ bezeichnet. Dieses Phänomen erzeugt metabolischen Stress in den Fettzellen und setzt mit der Produktion reaktiver Sauerstoffspezies (ROS) und freier Radikale eine Situation oxidativen Stresses in Gang [8]. In der Anfangsphase des Blebbing bleibt die Membran des Adipozyten intakt (die Fettzellen sind leer, und ihre Größe hat abgenommen), aber die kontinuierliche Bildung molekularer Spezies wie ROS und freie Radikale sowie die Beteiligung von Adipozytenlipasen [9] führen schließlich zur Auflösung der Zellmembran. Kurz gesagt, sind die beschädigten Adipozyten jene, bei denen das Phänomen des „Blebbing“ durch die direkte Wirkung der MW intensiver ist und so das Aufbrechen der Zellmembran stimuliert. Bei anderen Adipozyten mag das Blebbing mehr Zeit in Anspruch nehmen, aber wenn es intensiver und ausgedehnter stattfindet, führt es zu weiteren Zerstörungen der Zellmembranen, wobei deren Fettanteil ins Interstitium freigesetzt wird. Als Folge dieser strukturellen und funktionalen Ereignisse wird der Vorgang der zellulären Lipolyse aktiviert. Weiterhin muss berücksichtigt werden, dass selbst dann, wenn die Membran der Fettzelle nicht unmittelbar aufbricht, sie in den folgenden Wochen irreparable Schäden erleiden kann. Während dieser Vorgänge, die zur zellulären Lipolyse führen, wird die Migration von Makrophagen angeregt [10]. Die meisten Makrophagen lagern sich um die beschädigten Fettzellen an, wo sie charakteristische Strukturen, die sog. „crown like structures“ (CLS), bilden [11, 12]. Die Makrophagen werden durch einzelne Kontakte aktiviert und bilden dann umfangreiche vielkernige Synzytien, die nicht nur in den Abtransport von Fetttröpfchen (freie Fettsäuren, Cholesterin etc.), sondern auch von Zellfragmenten beschädigter Adipozyten eingreifen. Dieser Vorgang der Entfernung durch Makrophagen ist ein Verteidigungsmechanismus zur Wiederherstellung der physiologischen Homöostase.

## Materialien und Methoden

Neunzehn gesunde Patienten (im Alter von 24 bis 55 Jahren, was einem Altersdurchschnitt von 39 Jahren entspricht) – 10 Frauen und 9 Männer – mit sichtbaren Fettansammlungen im Bauchbereich erhielten 3 Mikrowellenbehandlungssitzungen (im Abstand von jeweils 4 Wochen) unter Verwendung des neuen Onda Plus Body Shaping-Systems (von DEKA, Florenz, Italien). Dieses Gerät verwendet einen Mikrowellenapplikator mit einer Frequenz von 2,45 GHz und einer integrierten Kühlung für einen optimalen Behandlungskomfort des Patienten während der Anwendung. Es erzeugt eine kontrollierte Abgabe von MW, die sowohl die oberen Hautschichten als auch die tief liegenden Muskeln und Organe schützt. Die MW werden selektiv, durch einen tiefen und lokalen Einsatz der Energie in der Hypodermis, in die subkutane Fettschicht abgegeben. Die Konstruktion des Handstücks ermöglicht es, potenzielle Verstreuungen des elektromagnetischen Felds einzuschränken. Die in dem Handstück integrierte permanente Kühlung bewahrt außerdem die oberflächlicheren Hautschichten vor unerwünschter Überhitzung. Das vorliegende System ist mit einem Shallow-Handstück (flach) ausgestattet, das in erster Linie zur Hautstraffung und für oberflächliche Cellulite ausgelegt ist, sowie einem Deep-Handstück (tief), das für Fettablagerungen und tiefe Cellulite geeignet ist. Das Shallow-Handstück bewirkt eine konzentriertere und oberflächliche Erwärmung und erhitzt das Gewebe fokussiert bis in eine Wirktiefe von bis zu 0,7 cm. Zusammen mit der integrierten Kühlung (bis 5 °C) erzeugt dieses Handstück eine kontrollierte Hyperthermie, die auf die Auflösung des fibrösen Kollagens und die Stimulation der Schrumpfung der oberflächlichsten Kollagenfasern abzielt, um sowohl straffend als auch remodellierend auf das oberflächliche Bindegewebe zu wirken. Das Deep-Handstück ist ausgelegt für die Erwärmung in eine Wirktiefe von bis zu 1,2 cm. Es erzeugt eine kontrollierte Hypothermie, die in den Adipozyten eine molekulare Schwingung bewirkt und die tieferen Kollagenfasern solubilisiert und somit die Lipolyse der Fettzellen sowie die Neumodellierung der Kollagenfasern durch Fibroblastenaktivierung einleitet. Um die Durchführung der Behandlung zu vereinfachen, ist an beiden Handstücken eine LED-Anzeige angebracht, die über die Kopplung der Applikatoren informiert.

Die Studie wurde unter Berücksichtigung der Deklaration von Helsinki durchgeführt. Vor Beginn der Behandlung haben alle Patienten eine Einwilligungserklärung sowie ein Fotofreigabeformular unterzeichnet. Es wurden eine vorläufige Untersuchung und eine Anamnese aller Patienten durchgeführt, um eine Auswahl der Patienten für die Studie zu treffen und um das für die Studie am besten geeignete Behandlungsprotokoll zu bestimmen. Zu diesem Zweck wurden Informationen über die Patientenhistorie erhoben, und es wurde eine Untersuchung durchgeführt. Ausschlusskriterien für die Behandlung lokaler Fettansammlungen waren: adipöse Patienten (definiert als BMI [Body Mass Index] > 30), Patienten mit einer großflächig über den gesamten Körper verteilten Fettschicht, Patienten mit einer begrenzten Dicke des Unterhautfettgewebes (< 1 cm bzw. einem ungefähren Pinch von < 2 cm). Darüber hinaus wurden von jeglicher Art der Behandlung ausgeschlossen: Patienten, die sich einer antikoagulativen oder antiaggregierenden Behandlung unterzogen (Möglichkeit dauerhafter Hautrötungen) oder systemisch/oral verabreichte Steroide erhielten, Patienten mit offenen Wunden im Behandlungsbereich, minderjährige Patienten, stillende/schwangere Patientinnen, Patienten mit unrealistischen Erwartungen. Weiterhin wurden Patienten für die Behandlung nicht zugelassen, wenn sie in den vergangenen 3 Monaten bereits eine Behandlung zur Fettreduktion oder gegen Cellulite oder Hautschlaffheit erhalten hatten.

Die Patienten wurden in 3 Gruppen, je nach der Größe des abdominalen Pinchwerts, eingeteilt. Gruppe 1 bestand aus 4 Patienten mit einem Pinch von mehr als 4,5 cm, Gruppe 2 bestand aus 10 Patienten mit einem Pinch von 2,5–4,5 cm, und Gruppe 3 bestand aus 5 Patienten mit einem Pinch von weniger als 2,5 cm. Alle Patienten wurden angemessen über das Behandlungsverfahren aufgeklärt sowie über die zu erwartenden Resultate im Hinblick auf ihre jeweiligen individuellen Charakteristika, die voraussichtliche Anzahl der für die gewünschten Ergebnisse erforderlichen Behandlungssitzungen und die vor und nach jeder Behandlung zu befolgenden Schritte und möglichen Nebenwirkungen. Des Weiteren wurden bei jedem Patienten bei der ersten Vorstellung (T0), 4 Wochen nach der 1. Behandlungssitzung (T1), 4 Wochen nach der 2. Behandlungssitzung (T2) und 4 Wochen nach der 3. Behandlungssitzung (T3) eine Umfangsmessung und eine fotografische Dokumentation durchgeführt. Mit dem Einverständnis der Patienten wurde vor der ersten Behandlungssitzung (T0) und 24 h nach der letzten Sitzung (T3) eine Bluttestdokumentation von jedem Patienten erstellt.

Vor jeder Behandlungssitzung wurden die Patienten gebeten, ihren Bauchbereich zu säubern und etwaige Verunreinigungen, die die MW beeinflussen oder die Flächen der Handstücke blockieren könnten, zu entfernen. Gegebenenfalls sollte dichte Behaarung in dem zu behandelnden Bereich rasiert werden, um die Kopplung zwischen dem Handstück und der Haut zu verbessern. In diesem Stadium wurde eine allgemeine Analyse des körperlichen Zustands des Patienten (in stehender Position) durchgeführt, um die zu behandelnden Bereiche besser zu identifizieren – sowohl in Bezug auf die Art als auch den Zustand der Unvollkommenheit/des Krankheitsbilds. Dann wurden bei dem ruhig stehenden Patienten 15 cm × 15 cm große Behandlungsbereiche bestimmt und mit einem hautverträglichen Stift gekennzeichnet, um später, wenn sich der Patient auf der Behandlungsliege befindet, die Behandlungsareale besser bestimmen zu können. Es wurde ein geeignetes Protokoll festgelegt, und die Behandlung wurde in jedem Bereich ca. 10 min lang durchgeführt [13].

Was das Protokoll angeht, wurden die Patienten der Gruppe 1 mit einer Energiedosis von 140 W, 100.000 J und mit dem Deep-Handstück behandelt. Die Patienten der Gruppe 2 wurden mit einer Energiedosis von 130 W, 80.000 J und ebenfalls dem Deep-Handstück behandelt. Und schließlich wurden die Patienten der 3. Gruppe mit einer Dosis von 120 W, bis zu 70.000 J und dem Shallow-Handstück behandelt. In allen Fällen lag die Kühlung bei 5 °C und die Behandlungszeit pro identifiziertem Bereich bei 7–10 min. Die Behandlung wurde mit kreisförmigen und linearen Bewegungen durchgeführt. Die Behandlungszeit lag (im Durchschnitt) bei ungefähr 20–40 min pro Patient.

Es wurden sowohl qualitative als auch quantitative Beurteilungen des Gewebes durchgeführt (Hautfalte dicker als 2 cm und dünner als 5,5 cm). Bei dem vorbereitenden Palpationsscreening wurden keine Knötchen, fibrotisches Gewebe oder andere Anomalien festgestellt, sodass die Behandlung eingeleitet werden konnte.

Sobald sich der Patient auf der Behandlungsliege befand, wurde ein dünner Film reinen Vaselineöls in pharmazeutischer Qualität über den gesamten Behandlungsbereich gesprüht, um einen einwandfreien Kontakt zwischen Handstück und Haut, bessere Kopplung und größere Beweglichkeit zu gewährleisten.

Nach der Behandlung wurde in dem behandelten Bereich eine Lymphmassage durchgeführt. Die allgemeinen Anweisungen für die Patienten lauten: eine angemessene gesunde Ernährung und moderate körperliche Aktivität.

## Ergebnisse

**Figure 1:**
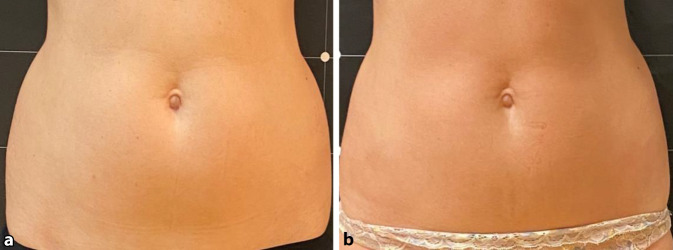

Zum Zeitpunkt T3 wurden klinisch messbare Ergebnisse im Bauchumfang statistisch festgestellt und mit statistischer Signifikanz berichtet (*p* < 0,001) (Abb. 1 und 2). Beim T1-Follow-up betrug der durchschnittliche Verlust bei allen Patienten 1,34 ± 0,78 cm. Eine weitere Reduzierung des Bauchumfangs von durchschnittlich 2,55 ± 1,10 cm wurde dann bei T2 dokumentiert. Zum Abschluss wurde in allen Gruppen ein Durchschnitt von 3,80 ± 1,21 cm festgestellt. Die Tab. 1 dokumentiert für jeden Patienten die Reduzierung des Bauchumfangs (cm) bei T1, T2 und T3.

**Figure 2:**
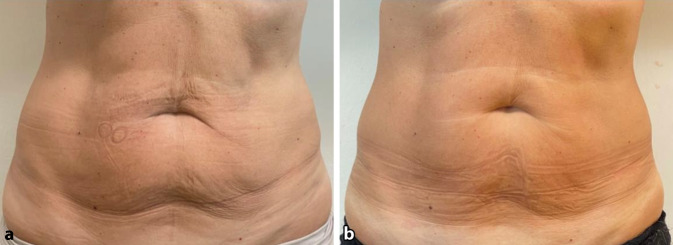

**Table 1:** 

Patienten-ID	Gruppe 1 (P > 4,5 cm)	Gruppe 2 (2,5 cm < P < 4,5 cm)	Gruppe 3 (P < 2,5 cm)	Geschlecht	Reduzierung des Bauchumfangs @ T1 [cm]	Reduzierung des Bauchumfangs @ T2 [cm]	Reduzierung des Bauchumfangs @ T3 [cm]
1			3	W	0,5	1,00	2,00
2		2		W	2,00	3,00	3,50
3		2		W	1,50	2,50	2,50
4	1			W	2,00	3,50	5,00
5		2		W	1,00	1,50	3,00
6	1			W	1,50	3,00	4,00
7		2		W	1,50	2,00	3,50
8	1			W	0,50	1,50	5,00
9		2		W	1,00	2,50	4,00
10		2		W	2,00	3,00	3,50
11		2		M	3,50	6,00	7,00
12			3	M	0,50	2,50	3,00
13	1			M	1,00	2,50	4,00
14			3	M	1,00	2,00	3,00
15		2		M	2,00	3,00	4,00
16			3	M	0,50	1,50	2,50
17		2		M	1,00	2,50	4,50
18			3	M	0,50	1,50	2,50
19		2		M	2,00	3,50	4,50
*Insgesamt*	4	10	5				

Es wurde weder während noch nach der Behandlung ein besonders geröteter Bereich festgestellt.

Alle 19 Patienten wurden per Blutuntersuchung überwacht. Die überwachten Patienten zeigten nach der letzten Behandlung keine signifikanten Veränderungen in ihren Blutwerten (Triglyceride und Cholesterin). Es wurde kein erheblicher Anstieg der Transaminasen festgestellt, was bedeutet, dass die Leberfunktion unbeeinträchtigt war. Weiterhin wurde keine Veränderung der Muskulatur festgestellt, und auch die Erkennungsindizes für die Nierenfunktion blieben während des gesamten Behandlungsprotokolls stabil.

## Diskussion

Unsere Studie hat in Übereinstimmung mit der Literatur zu dieser Technologie die Effizienz und Sicherheit von Mikrowellen für die Behandlung lokaler Fettablagerungen nachgewiesen. Eine erste an einer Gruppe von 12 Freiwilligen mit lokalisierten Fettablagerungen durchgeführte Pilotstudie [14] hatte über die vielversprechende Rolle von Mikrowellen bei der Behandlung lokalisierter subkutaner Fettpolster berichtet. Im Verlauf der MW-Behandlungen stellten die Patienten mit Fettablagerungen im Bauchbereich eine schrittweise Verringerung ihres Bauchumfangs fest. Vier Wochen nach der letzten Behandlungssitzung wurde eine mittlere Reduktion von 3,90 cm dokumentiert. Bei der Auswertung der Blutuntersuchungen hatte sich keiner der Werte signifikant verändert. Sämtliche Resultate stimmen mit unseren Ergebnissen überein. Darüber hinaus erklärten die Patienten übereinstimmend, dass sie mit den Behandlungsergebnissen zufrieden waren. Es wurden keine unerwünschten Nebenwirkungen berichtet. Eine weitere Studie [15] mit 49 Teilnehmern beurteilte die Wirkung der Mikrowellentechnologie auf Bauchfett sowie die anthropometrischen Indizes übergewichtiger Erwachsener und gelangte zu dem Schluss, dass dieses Verfahren eine deutlich reduzierende Auswirkung auf die Kennwerte der anthropometrischen Indizes hat. Daher wird sie als eine sichere und effektive Methode für die Verbesserung des Hautbilds und die Reduzierung von subkutanem Fett betrachtet.

Mikrowellentechnologie verbessert das Hautbild und reduziert subkutanes Fett

Ein wichtiges Merkmal dieser Technologie ist, dass ihre Effektivität nicht auf die Wirkung auf subkutanes Fett beschränkt ist, sondern auch bei Hautschlaffheit und Cellulite wirkt. Eine jüngere Studie [16] beurteilte die Sicherheit und Wirksamkeit von MW in der Behandlung von submentaler Hautschlaffheit und Fett bei 48 erwachsenen Probanden. Sechs Monate nach der letzten Behandlung hatten sich beim Follow-up alle untersuchten Parameter zur Beurteilung des submentalen Fetts, der submentalen Hautschlaffheit und der Schmerzen verbessert und bestätigten somit die Wirksamkeit und Sicherheit des Systems auch für die submentale Region.

Zerbinati et al. [17] entdeckten in einem Tiermodell neue morphologische Beweise für strukturelle Veränderungen in kompaktem Kollagen, die interlobuläre Septen im Unterhautfettgewebe bilden. Nach einer MW-Behandlung mittels Pikro-Siriusrot-Färbung und zirkularer Polarisationsmikroskopie erhobene morphologische und morphometrische Daten haben gezeigt, dass verdichtete Kollagenfasern, welche die fibrotischen Septen bilden, wenige Stunden nach einer MW-Behandlung beginnen, zu fragmentieren und sich umzubauen.

Bonan et al. [18] beurteilten anhand einer Gruppe von 15 weiblichen Probanden die Wirksamkeit und Sicherheit von Mikrowellen bei Cellulite (ödematöse fibrosklerotische Pannikulopathie [EEP]) und Hautschlaffheit im Bereich von Oberschenkeln und Gesäß. Die Studie gab an, dass es 8 Wochen nach der letzten Behandlungssitzung eine signifikante Verbesserung sowohl in der Ausprägung der Cellulite als auch bei der Hautschlaffheit gab. Es wurden keine Schmerzen oder Nebenwirkungen berichtet oder festgestellt. Anhand einer Skala zur Patientenzufriedenheit wurde eine generelle Zufriedenheit dokumentiert. Diese ersten Ergebnisse deuten darauf hin, dass das Onda Plus-System eine sichere, effektive und gut verträgliche Behandlungsmethode bei Cellulite und Hautschlaffheit ist, selbst für Patienten mit geringer körperlicher Betätigung oder für Raucher (90 % bzw. 40 %).

Es unterzogen sich 26 Frauen mit schwerwiegender oder moderater Cellulite 4 MW-Therapie-Behandlungssitzungen an ihrem Gesäß und ihren Oberschenkeln [19]. Die Ergebnisse der Cellulite-Schweregrad-Skala (CSS) sowie der Klassifizierung nach Nürnberger-Müller ergaben beide, dass die Behandlung eine positive Wirkung auf den Schweregrad der Cellulite hatte.

Nisticò et al. [20] untersuchten die Wirksamkeit kombinierter MW und FMS („flat magnetic stimulation“) bei der Behandlung lokaler Fettansammlungen im Bauchbereich und Hautschlaffheit. Dabei wandten sie ein synergetisches Vorgehen an Binde- und Muskelgewebe an, das auf ihrer Erfahrung mit der Wirksamkeit von MW bei subkutanem Fett und Hautschlaffheit und einer neuen elektromagnetischen Stimulationstechnik, der vorgenannten FMS, basierte. Es waren 25 Patienten registriert. Die Ergebnisse zeigen, dass die Kombination von MW und FMS-Behandlung sicher und wirksam für die Behandlung von subkutanem Bauchfett und Hautschlaffheit ist.

## Fazit für die Praxis

- Diese klinische Evaluierung zeigt mit statistischer Signifikanz eine nachhaltige Umfangsreduzierung sowie eine Verbesserung im Erscheinungsbild des behandelten Bauchbereichs nach der Behandlung mit dem MW(Mikrowellen)-Body-Contouring-System gemäß den Kriterien, welche die Literatur für diese Anwendungsart vorgibt.
- Dieses Gerät hat sich als ein sicheres und effektives Instrument für die Volumenverringerung von Fettgewebe bei jedem behandelten Hauttyp und unter Berücksichtigung bestehender Technologien erwiesen.
- Bei keiner Behandlungssitzung wurde über Schmerzen oder spürbares Unbehagen berichtet.
